# Weighted Lin-Wang Tests for Crossing Hazards

**DOI:** 10.1155/2014/643457

**Published:** 2014-03-30

**Authors:** James A. Koziol, Zhenyu Jia

**Affiliations:** ^1^College of Health, Human Services and Science, Ashford University, San Diego, CA 92128, USA; ^2^Department of Statistics, University of Akron, Akron, OH 44325, USA; ^3^Department of Family and Community Medicine, Northeast Ohio Medical University, Rootstown, OH 44272, USA; ^4^Guizhou Provincial Key Laboratory of Computational Nano-Material Science, Guizhou Normal College, Guiyang 550018, China

## Abstract

Lin and Wang have introduced a quadratic version of the logrank test, appropriate for situations in which the underlying survival distributions may cross. In this note, we generalize the Lin-Wang procedure to incorporate weights and investigate the performance of Lin and Wang's test and weighted versions in various scenarios. We find that weighting does increase statistical power in certain situations; however, none of the procedures was dominant under every scenario.

## 1. Introduction 

Lin and Wang [1] have recently introduced an ingenious modification of the two-sample logrank statistic, appropriate for crossing hazards alternatives. Through a simulation study, they demonstrated that their modified test had greater power than the commonly used logrank and Wilcoxon tests for detecting differences between crossing survival curves. In this note, we propose weighted versions of the Lin-Wang (LW) test and investigate the performance of these weighted tests in a limited simulation study. Details are given in Section 2, and the simulation results are presented in Section 3. We give an example in Section 4 and conclude remarks in Section 5.

## 2. Methods 

For consistency, we adhere to the notational conventions introduced by Lin and Wang [1]. We have survival data from two groups of subjects, the groups being labeled I and II, and are interested in comparing the survival distributions of the two groups. Events (failures or deaths) are observed at *r* distinct time points  *t*_1_ < ⋯<*t*_*r*_ across the pooled groups. At time *t*_*j*_, the number of observed failures in each of the two groups is denoted by *d*_1*j*_ for Group I and *d*_2*j*_ for Group II, and the numbers at risk just before time *t*_*j*_ are denoted by *n*_1*j*_ and *n*_2*j*_, respectively, for *j* = 1,2,…, *r*. Consequently, at time *t*_*j*_, there are *d*_*j*_ = *d*_1*j*_ + *d*_2*j*_ failures out of *n*_*j*_ = *n*_1*j*_ + *n*_2*j*_ subjects. Subjects may be censored during or at the end of the period of observation. A representative 2 × 2 contingency table of group by status at observed failure time *t*_*j*_ is given in Table 1.

We are interested in assessing the null hypothesis

 
*H*_0_: the survival distributions of the two groups are identical  versus the global alternative hypothesis.
 
*H*_1_: the survival distributions of the two groups are not identical.

Lin and Wang introduced the quadratic statistic

(1) $$\begin{array}{c} \Delta =\overset{r}{\underset{j=1}{\sum }}{\left[d_{1j}-E\left(d_{1j}\right)\right]}^{2} \end{array}$$

for comparison of the two groups: they argued that Δ reflects the quadratic distance between the two underlying survival distributions hence should be sensitive to differences in either direction. They therefore based inference relating to *H*_0  _on the standardized version of Δ, which they denoted as T*. 

Let us define a weighted version of Δ as

(2) $$\begin{array}{c} \Delta_{w}=\overset{r}{\underset{j=1}{\sum }}w_{j}*{\left[d_{1j}-E(d_{1j})\right]}^{2} \end{array}$$

with arbitrary weights *w*_*j*_, usually nonnegative. Our test statistic for assessing *H*_0  _is the standardized version of Δ_*W*_; namely,

(3) $$\begin{array}{c} T_{w}=\frac{\Delta_{w}-E\left(\Delta_{w}\right)}{\sqrt{\mathrm{Var}\left(\Delta_{w}\right)}}, \end{array}$$

where *E*(Δ_*W*_) and Var⁡(Δ_*W*_) are calculated from the marginal hypergeometric distribution of the *d*_1*j*_. In particular,

(4) $$\begin{array}{c} E\left(\Delta_{w}\right)=\overset{r}{\underset{j=1}{\sum }}w_{j}*\frac{n_{1j}n_{2j}d_{j}\left(n_{j}-d_{j}\right)}{n_{j}^{2}\left(n_{j}-1\right)}, \end{array}$$

and Var⁡(Δ_*W*_) is given by

(5) Var⁡(Δw)=∑j=1rwj2∗{E(d1j4)−4E(d1j3)E(d1j)+6E(d1j2)[E(d1j)]2−3[E(d1j)]4−[Var⁡(d1j)]2}.

The raw moments of *d*_*ij*_ can be readily calculated from the following expression for the factorial moments:

(6) $$\begin{array}{c} E\left(d_{1j}^{\left(r\right)}\right)=\frac{n_{1j}^{\left(r\right)}d_{j}^{\left(r\right)}}{n_{j}^{\left(r\right)}}, \end{array}$$

where *n* ^(*r*)^ = n*(*n* − 1)∗ ⋯ ∗(*n* − *r* + 1). For reference,

(7) $$\begin{array}{c} E\left(d_{1j}\right)=\frac{n_{1j}d_{j}}{n_{j}}, \end{array}$$

(8) $$\begin{array}{c} \mathrm{Var}\left(d_{1j}\right)=\frac{n_{1j}n_{2j}d_{j}\left(n_{j}-d_{j}\right)}{n_{j}^{2}\left(n_{j}-1\right)}, \end{array}$$

(9) $$\begin{array}{c} E\left(d_{1j}^{2}\right)=\mathrm{Var}\left(d_{1j}\right)+{\left[E(d_{1j})\right]}^{2}, \end{array}$$

(10) $$\begin{array}{c} E\left(d_{1j}^{3}\right)=3E\left(d_{1j}^{2}\right)-2E\left(d_{1j}\right)+\frac{n_{1j}^{\left(3\right)}d_{j}^{\left(3\right)}}{n_{j}^{\left(3\right)}}, \end{array}$$

(11) $$\begin{array}{c} E\left(d_{1j}^{4}\right)=6E\left(d_{1j}^{3}\right)-11E\left(d_{1j}^{2}\right)+6E\left(d_{1j}\right)+\frac{n_{1j}^{\left(4\right)}d_{j}^{\left(4\right)}}{n_{j}^{\left(4\right)}}. \end{array}$$

We note in passing that there are typographical errors in the expressions for *E*(*d*_*ij*_^3^) and *E*(*d*_*ij*_^4^) in Lin and Wang [1].

Under the same assumptions as enumerated by Lin and Wang [1]; namely, the underlying failure times are independent, the censoring distributions (if any) for group I and group II are independent of each other, and of the respective survival distributions, the total number of observed failures and the distinct number of failure times are large, and the weights are positive and bounded; then *T*_*w*_ approximately follows a standard normal distribution. We are thus specifying the usual random censorship model, with further conditions to ensure approximate normality of *T*_*w*_. For assessing the null hypothesis of equality of the underlying survival distributions of the two groups, Lin and Wang propose a two-sided test statistic based on T*, and we will follow that convention with *T*_*w*_.

## 3. Simulation Studies 

In this section, we will investigate the empirical performance of weighted versions of the LW statistic, compared to the original (unweighted) LW statistic.

### 3.1. Empirical Type I Error

We first investigate achieved significance levels of the LW statistic and three weighted versions. Following LW, we generated two independent random samples from the exponential distribution with mean of 4. The censoring distribution is Uniform (0, 20) in each group. The number of iterations in each simulation study is 5000. The empirical Type I error is calculated as the proportion of 5000 repeated random samples in which we reject the null hypothesis at the alpha = 0.05 significance level, under the assumption that *T* and weighted versions *T*_*w*_ have normal distributions, and two-sided tests are utilized. We report on three weighted versions of the LW statistic, delineated by different sets of weights *w*_*j*_,  1 ≤ *j* ≤ *r*: (i) *w*_*j*_ = *n*_*j*_; (ii) *w*_*j*_ = √*n*_*j*_; (iii) *w*_*j*_ = 1/SD(*d*_1*j*_), where SD(*d*_1*j*_) = √Var⁡(*d*_1*j*_). The empirical Type I errors are given in Table 2.

In this limited simulation study the empirical Type I errors are quite close to the theoretical 0.05 value, for both the LW statistic and the weighted variants. The normal distribution seems an adequate approximation for the sample sizes investigated.

### 3.2. Empirical Power

Following LW, we undertook simulation studies comparing the empirical powers of the unweighted LW statistic with its weighted variants, under the three following scenarios.

**Scenario 1**

This scenario entails crossing survival curves. The LW specification is as follows. “In Group I the survival times follow an exponential distribution with mean of 6. In Group II the survival times follow an exponential distribution with mean of 2. However, if the survival time in Group II is greater than or equal to 1.5, then the survival time is regenerated to follow an exponential distribution with mean of 40. The censoring distribution is Uniform (0, 20) in Group I and Uniform (0, 100) in Group II, which result in about 24% censoring rate in Group I and 18% in Group II, respectively.”

**Scenario 2**

In this situation, the two survival curves are initially close, then cross, and diverge. The LW description is as follows. “In Group I the survival times follow an exponential distribution with mean of 4. In Group II the survival times follow an exponential distribution with mean of 3. However, if the survival time in Group II is greater than or equal to 4, then the survival time is regenerated to follow an exponential distribution with mean of 20. Also, censoring is assumed to occur randomly across the two groups. For each subject in the two groups, an independent Uniform (0, 1) random variable *U* is generated. In Group I, if *U* is less than 0.2, then the corresponding time point will be flagged as censored. Otherwise it is not censored. The censoring in Group II is created similarly but with a different rate. The censoring rate is 20% in Group I and 30% in Group II, respectively.”

**Scenario 3**

Here, the proportional hazards assumption obtains. The LW specification is as follows. “The survival times follow an exponential distribution with means 2 and 5 in Groups I and II, respectively. The censoring mechanism is similar to that in Situation (Scenario 2), but this time with 20% censoring rate in Group I and 15% censoring rate in Group II, respectively.”

The number of iterations in each simulation study is 5000. The estimated statistical power is calculated as the proportion of 5000 repeated random samples in which we reject the null hypothesis at the nominal alpha = 0.05 significance level, with two-sided test statistics. The weighted versions of the LW statistic are as above, namely, (i) *w*_*j*_ = *n*_*j*_; (ii) *w*_*j*_ = √*n*_*j*_; (iii) *w*_*j*_ = 1/SD(*d*_1__*j*_), where SD(*d*_1*j*_) = √Var⁡(*d*_1*j*_). Findings for the three scenarios are given in Tables 3, 4, and 5, respectively.

Interestingly, none of the procedures is dominant under every scenario. We might tend to favor the LW statistic under Scenario 1, the weighted version LW_*w*3_ under Scenario 2, and the weighted versions LW_*w*1_ and LW_*w*2_ under Scenario 3.

## 4. An Example

**Figure 1:**
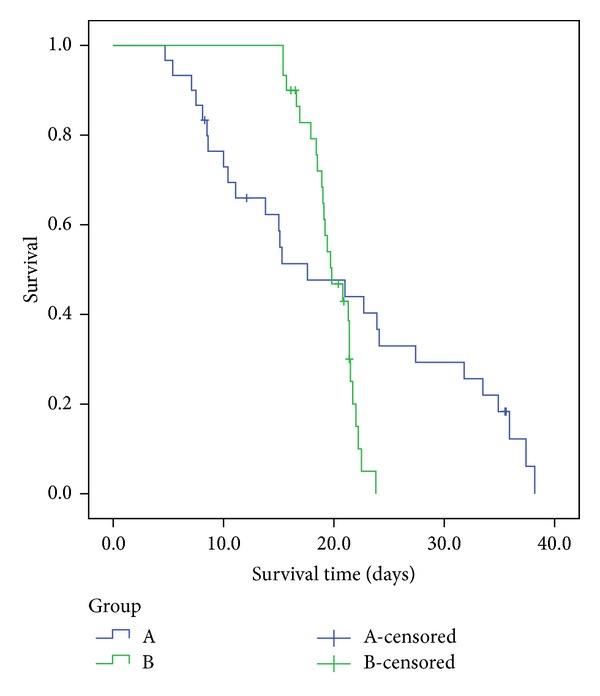
Kaplan-Meier survival curves for two groups of leukemic mice. Initial sample sizes were 30 per group. There were 4 censored observations in Group (A) and 5 in Group (B).

We will apply the various procedures to data arising from a cancer chemotherapy experiment, as explained in Koziol [2] and Koziol and Yuh [3]. Briefly, sixty leukemic mice were randomly subdivided into two groups of equal size; one group (Group (a)) was treated with a new investigative chemotherapeutic agent, and the other group (Group (b)) served as controls. Survival times of the two cohorts are given in Table 6, and Kaplan-Meier survival curves for the groups are depicted in Figure 1.

Clearly, we are in crossing hazards setting, and the logrank test and the generalized Wilcoxon test are not necessarily sensitive to this type of alternative. Indeed, with these data, the logrank chi-square statistic (with 1 d.f.) is 1.36 (*P*  =  0.24), and the generalized Wilcoxon chi-square statistic is 1.12 (*P*  =  0.27); we would fail to reject the hypothesis of equality of survival distributions for the two cohorts with either of these tests.

On the other hand, the LW statistic and its weighted variants all point to significantly different survival experiences in the two cohorts, with *P* values of 10^−6^ or smaller. In comparison, the omnibus Kolmogorov-Smirnov, Kuiper, and Cramér-von Mises statistics introduced by Koziol and Yuh [3] were also indicative of significantly different survival distributions but with more modest *P* values of 10^−3^.

## 5. Concluding Remarks 

The logrank test as described in Section 2 should be ascribed to Mantel [4]: Mantel brilliantly intuited that the Mantel-Haenszel (MH) statistic [5] for assessing association across independent 2 × 2 tables could be applied to survival data, by constructing a 2 × 2 table as in Table 1 at each event (death) time then combining the resulting 2 × 2 tables as in the MH procedure.

Correspondingly, our incorporation of weights into the LW statistic as described in Section 2 is not new: our motivation devolves from similar introduction of weights into the Mantel formulation of the logrank statistic, by Tarone and Ware [6] and Leurgans [7] among others. And, anticipating the findings in Section 3, these investigators have shown that the weights can enjoy improved power properties over the unweighted MH statistics in various settings. We remark that calculation of the LW statistic is rather computationally intensive; but incorporation of weights should cause no additional computational difficulties. Optimal choice of weights remains an open issue, which we are currently pursuing.

The generalized Wilcoxon test and the logrank test are perhaps the best known and most commonly used procedures for the comparison of two survival distributions with observations subject to random censorship. Mantel [4] and others recognized, however, that these tests may not be appropriate whenever the alternative of interest is not that the one survival distribution is stochastically larger than the other but merely that the distributions are not equal. Crossing hazards are an example of nonstochastic ordering of survival distributions. For testing equality against such alternatives, Koziol [2] proposed a two-sample Cramér-von Mises type statistic based on the product-limit estimates of the individual survival distributions, and later Koziol and Yuh [3] introduced Kolmogorov-Smirnov and Kuiper as well as Cramér-von Mises statistics for the same omnibus two-sample testing problem. The LW statistic is more closely attuned to the logrank test than these omnibus procedures; and, as seen in the example, the LW statistics may be more sensitive to crossing hazards alternatives.

It should be noted that Mantel [4] also proposed a modification of the Mantel logrank test, appropriate for crossing hazards: Mantel suggested that one construct a “chi-squared” statistic at each event time as in Table 1, sum these individual statistics over the event times, and then treat the resulting sum as an approximate chi-square random variable with *n* degrees of freedom, *n* being the number of tables (distinct event times). We explored this statistic in simulation studies, but regrettably we cannot recommend this statistic, due to decreased power relative to the other statistics reported herein, and the tenuous assumption that a chi-square distribution for this statistic is adequate (though with larger sample sizes, a normal approximation might be invoked).

## Figures and Tables

**Table 1:** Survival experience of the two groups at observed failure time *t*_*j*_.

Group	Number of failures	Number of non-failures	Number at risk just before *t*_*j*_
I	*d*_1*j*_	*n*_1*j*_ − *d*_1*j*_	*n*_1*j*_
II	*d*_2*j*_	*n*_2*j*_ − *d*_2*j*_	*n*_2*j*_

Total	*d*_*j*_	*n*_*j*_ − *d*_*j*_	*n*_*j*_

**Table 2:** Empirical levels of the Lin-Wang test, and three weighted variants.

Sample sizes	LW	LW_*w*_1__	LW_*w*_2__	LW_*w*_3__
(20,20)	0.044	0.045	0.044	0.042
(30,30)	0.048	0.053	0.053	0.048
(40,40)	0.046	0.048	0.047	0.045
(50,50)	0.051	0.053	0.050	0.049
(60,60)	0.053	0.045	0.051	0.053
(70,70)	0.049	0.049	0.051	0.047
(80,80)	0.056	0.047	0.053	0.053
(90,90)	0.051	0.049	0.050	0.050
(100,100)	0.048	0.050	0.049	0.048

Notes: Sample sizes are given for group 1, followed by group 2. LW denotes the Lin-Wang test, and LW_*w*_*i*__ denotes the weighted version of the LW test, with weights *w*_*i*_ as described in the text. The underlying distributions of group 1 and group 2 were identical, as described in the text. The empirical levels of the two-sided test statistics were estimated from 5000 simulations, at nominal alpha level 0.05.

**Table 3:** Empirical powers of the Lin-Wang test, and three weighted variants, under Scenario 1.

Sample sizes	LW	LW_*w*_1__	LW_*w*_2__	LW_*w*_3__
(20,20)	0.406	0.269	0.331	0.407
(30,30)	0.627	0.524	0.589	0.602
(40,40)	0.813	0.772	0.81	0.774
(50,50)	0.904	0.902	0.912	0.87
(60,60)	0.952	0.968	0.965	0.922
(70,70)	0.982	0.992	0.99	0.965
(80,80)	0.99	0.998	0.996	0.981

Notes: Sample sizes are given for group 1, followed by group 2. LW denotes the Lin-Wang test, and LW_*w*_*i*__ denotes the weighted version of the LW test, with weights *w*_*i*_ as described in the text. The empirical powers of the two-sided test statistics were estimated from 5000 simulations, at nominal alpha level 0.05.

**Table 4:** Empirical powers of the Lin-Wang test, and three weighted variants, under Scenario 2.

Sample sizes	LW	LW_*w*_1__	LW_*w*_2__	LW_*w*_3__
(20,20)	0.089	0.039	0.053	0.097
(30,30)	0.157	0.046	0.084	0.167
(40,40)	0.222	0.057	0.116	0.239
(50,50)	0.314	0.077	0.169	0.339
(60,60)	0.402	0.106	0.225	0.433
(70,70)	0.484	0.122	0.273	0.511
(80,80)	0.549	0.151	0.338	0.583
(90,90)	0.612	0.173	0.379	0.644
(100,100)	0.675	0.212	0.431	0.700

Notes: Sample sizes are given for group 1, followed by group 2. LW denotes the Lin-Wang test, and LW_*w*_*i*__ denotes the weighted version of the LW test, with weights *w*_*i*_ as described in the text. The empirical powers of the two-sided test statistics were estimated from 5000 simulations, at nominal alpha level 0.05.

**Table 5:** Empirical powers of the Lin-Wang test, and three weighted variants, under Scenario 3.

Sample sizes	LW	LW_*w*_1__	LW_*w*_2__	LW_*w*_3__
(20,20)	0.433	0.430	0.435	0.387
(30,30)	0.600	0.630	0.628	0.532
(40,40)	0.728	0.775	0.767	0.647
(50,50)	0.822	0.878	0.872	0.753
(60,60)	0.889	0.929	0.925	0.827
(70,70)	0.931	0.970	0.962	0.884
(80,80)	0.964	0.985	0.982	0.933

Notes: Sample sizes are given for group 1, followed by group 2. LW denotes the Lin-Wang test, and LW_*w*_*i*__ denotes the weighted version of the LW test, with weights *w*_*i*_ as described in the text. The empirical powers of the two-sided test statistics were estimated from 5000 simulations, at nominal alpha level 0.05.

**Table 6:** The clinical data for sixty leukemic mice which were randomly subdivided into two groups (Group A and Group B) of equal size. “1” indicates the censored data.

Group A		Group B	
Survival (days)	Censoring	Survival (days)	Censoring
4.7	0	15.4	0
5.4	0	15.4	0
7.1	0	15.7	0
7.5	0	16.1	1
8.1	0	16.5	1
8.3	1	16.6	0
8.5	0	16.9	0
8.6	0	17.9	0
10	0	18.4	0
10.4	0	18.5	0
11.1	0	18.9	0
12.1	1	19	0
13.8	0	19.1	0
15	0	19.2	0
15.1	0	19.4	0
15.3	0	19.7	0
17.6	0	19.8	0
21	0	20.4	1
22.7	0	20.8	0
23.9	0	20.9	1
24.1	0	21.3	0
27.4	0	21.4	0
31.8	0	21.4	0
33.5	0	21.4	1
34.9	0	21.5	0
35.5	1	21.7	0
35.6	1	22	0
35.9	0	22.2	0
37.4	0	22.5	0
38.2	0	23.8	0
